# Low expression of CD39 on monocytes predicts poor survival in sepsis patients

**DOI:** 10.1186/s40560-025-00784-0

**Published:** 2025-03-10

**Authors:** Hangyang Li, Peili Ding, Yuyu Nan, Zhenping Wu, Ning Hua, Lixi Luo, Qinghua Ji, Fangfang Huang, Guobin Wang, Hongliu Cai, Saiping Jiang, Wenqiao Yu

**Affiliations:** 1 Department of Critical Care Medicine, The First Affiliated Hospital, Zhejiang University School of Medicine, 79 Qingchun Road, Hangzhou, 310003 China; 2 Department of Clinical Pharmacy, The First Affiliated Hospital, Zhejiang University School of Medicine, Hangzhou, China; 3 Department of Surgical Oncology, Zhejiang University School of Medicine Sir Run Run Shaw Hospital, Hangzhou, China; 4 Zhejiang Puluoting Health Technology Co., Ltd., Hangzhou, China

**Keywords:** Sepsis, Biomarkers, Monocytic CD39, Prognosis, Diagnosis

## Abstract

**Background:**

Sepsis is a critical condition associated with high morbidity and mortality, emphasizing the need for reliable biomarkers for its diagnosis and prognosis. This study uses advanced immunological techniques to evaluate monocytic CD39 (mCD39) expression as a potential marker in sepsis.

**Methods:**

This prospective observational cohort study included 206 participants from the First Affiliated Hospital, Zhejiang University School of Medicine between April 2022 and September 2023. Participants were categorized into four groups: healthy donors, patients with mild infections, post-cardiac surgery patients (non-infectious inflammation), and sepsis patients. Peripheral Blood Mononuclear Cells were analyzed using mass cytometry time-of-flight (CyTOF) with a 42-marker immune panel and flow cytometry targeting monocytes. Statistical analyses included ROC curves for diagnostic and prognostic performance and Kaplan–Meier survival analysis for prognostic evaluation.

**Results:**

Sepsis patients exhibited significantly lower monocytic CD39 expression than mild infection and post-surgery groups (*p* < 0.05). The diagnostic performance analysis revealed that mCD39 effectively distinguished sepsis from mild infection (AUC = 0.877) and non-infectious inflammation (AUC = 0.935). Prognostic analysis identified low mCD39 expression as a strong predictor of short-term survival, with a 7-day survival AUC of 0.85 (*p* = 0.037). Kaplan–Meier analysis showed that sepsis patients with low mCD39 expression had significantly lower 28-day survival rates (56.7% vs. 80.6%, *p* = 0.016).

**Conclusions:**

Low CD39 expression on monocytes might serve as a potential diagnostic biomarker and a strong predictor of poor prognosis in sepsis patients.

**Supplementary Information:**

The online version contains supplementary material available at 10.1186/s40560-025-00784-0.

## Background

Sepsis is one of the most severe pathological conditions in critical care patients, which represents dramatic immune response to infections and often leads to multi-organ dysfunctions [1, 2]. The prevalence of sepsis is on the rise and has become an important global healthcare issue [3, 4]. Early prediction of sepsis is crucial to the reduction of sepsis-related mortality [5, 6].

Sepsis was once characterized as the amalgamation of infection and Systemic Inflammatory Response Syndrome (SIRS) more than three decades ago [7]. However, the high sensitivity and relatively low specificity of the SIRS criteria made it insufficient to differentiate severe cases among patients with mild infection [1]. With the implementation of the Sepsis-3 criteria, Sequential Organ Failure Assessment (SOFA) scores have replaced SIRS as the primary tool for diagnosing sepsis [8]. Currently, the identification of sepsis still heavily relies on clinical assessment. Commonly used sepsis-related markers in clinical practice include white blood cell count (WBC), C-reactive protein (CRP), and procalcitonin (PCT). The limitation of current markers mainly lies in the lack of specificity in distinguishing sepsis from non-infectious inflammation or other pathological conditions [9–11].

The Surviving Sepsis Campaign and the latest Sepsis-3 definition underscore the necessity of reliable biomarkers that have the capability to classify septic patients into discrete biological phenotypes, such as hyperinflammatory or immunosuppressive subtype [8, 12, 13]. At the early stage of sepsis, the immune system, especially innate immune system, plays a pivotal role [14]. Therefore, it is crucial to find a specific and sensitive immune-related biomarker for sepsis.

Utilization of mass cytometry time-of-flight (CyTOF) in the immunology framework demonstrates notable efficacy in elucidating distinct cellular status and discovering novel markers specific to diseases [15–17]. In a recent study, researchers employed CyTOF technology to classify neutrophils in blood samples obtained from sepsis patients and identified two previously unrecognized subsets [18]. However, the study did not include other immune cells, such as monocytes, T cells, or B cells. Additionally, the comparison was made between the sepsis group and the non-infected inflammation group, while patients with mild infection who did not meet the Sepsis-3 criteria were not included.

In this study, we used CyTOF technology to conduct a thorough examination of Peripheral Blood Mononuclear Cells (PBMCs) using a panel of 42 immune-related markers for patients from four distinct groups: a healthy donor group, a mild infection group that did not meet the Sepsis-3 criteria, a group of patients after cardiac surgeries to represent non-infected inflammation condition, and a sepsis group. Furthermore, we investigated the differential expressions of CD39 on monocytes among different groups, and explored its prognostic role in predicting the survival outcomes of sepsis patients.

## Methods

### Study design

**Fig. 1:**
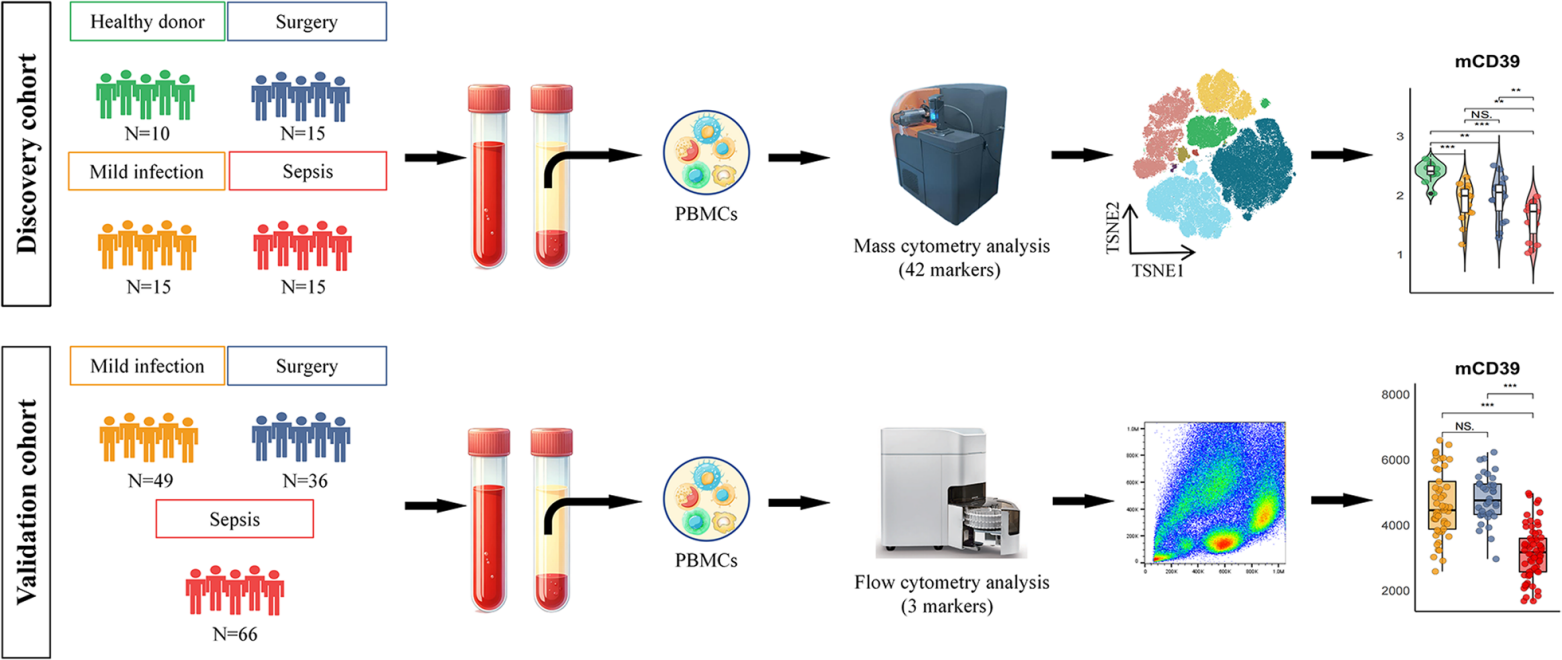
Workflow chart. The discovery cohort of this study consisted of blood samples obtained from healthy donors, patients with mild infection, patients with non-infectious inflammation who underwent cardiac surgeries, and patients with sepsis. Immunostainings targeting 42 markers were conducted and analyzed using mass cytometry in the discovery cohort. The validation cohort consisted of mild infection cases, post-cardiac surgery patients, and sepsis patients. The validation of 3 selected markers was conducted by the flow cytometry method. *PBMCs* peripheral blood mononuclear cells. *mCD39* monocytic CD39

This study was a prospective observational cohort study conducted at the First Affiliated Hospital, Zhejiang University School of Medicine (Hangzhou, China), between April 2022 and September 2023. A total of 206 subjects were recruited. Two cohorts were set, namely, the discovery cohort and the validation cohort and the workflow is illustrated in Fig. 1. We characterized our patients into 4 distinct subgroups: (1) a group of healthy donors; (2) patients with mild infection that did not meet the Sepsis-3 criteria; (3) post-operative patients who underwent cardiac surgery to represent the non-infectious inflammation condition; and (4) patients diagnosed as sepsis. The discovery cohort included 10 healthy donors, 15 patients with mild infection, 15 patients after cardiac surgery and 15 patients with sepsis. On the other hand, the validation cohort consisted of 49 patients with mild infection, 36 post-cardiac surgery patients, and 66 sepsis patients. Blood samples were obtained within 48 h of infection diagnosis, or following cardiac surgery. All human blood samples in this study were obtained with the approval of the donors (or their relatives) who provided written informed consent. This study was approved by the Clinical Research Ethics Committee of the First Affiliated Hospital, Zhejiang University School of Medicine (IIT20220076B).

### Inclusion and exclusion criteria

The inclusion criteria for this study were as follows:

Participants of 18 years or older were recruited. Patients in mild infection group should be: (1) diagnosed as infectious cases and (2) with an increase of SOFA score < 2 (Not compliant with Sepsis-3 criteria). Patients in sepsis group should be: (1) diagnosed as infectious cases and (2) with an increase of SOFA score ≥ 2 (In line with Sepsis-3 criteria). Patients in surgery group should be included within 24 h after cardiac surgery.

The exclusion criteria for this study were as follows:

(1) patients who have used glucocorticoids within the previous 6 months; (2) pregnant or lactating women; (3) cancer patients; (4) patients with hematologic disorders; and (5) patients with immunodeficiency, such as human immunodeficiency virus (HIV).

To avoid the influence or bias caused by perioperative infection and postoperative complications, cardiac surgery patients with nosocomial infections or complications should be excluded from the surgery group.

### Sample collection and processing

Peripheral blood samples (5 mL) were collected, and PBMCs were isolated using Ficoll gradient centrifugation. Cells underwent quality control, cryopreservation, and recovery processes for subsequent analyses. For detailed procedures, refer to the Supplementary Methods section.

### CyTOF and flow cytometry analyses

CyTOF analysis was performed using a panel of 42 immune-related markers. Cells were stained, fixed, and analyzed on a CyTOF system. Flow cytometry used a three-marker panel focusing on CD14 + monocytes. Samples underwent Fc receptor blocking and antibody incubation, followed by extensive washing and flow cytometer analysis. The process is detailed in the Supplementary Methods section.

### Statistics analysis

The data were summarized as mean ± standard deviation or median and inter-quartile range. Independent-sample *t* tests were employed for variables that followed a normal distribution, while the Wilcoxon rank-sum test was used for variables that did not adhere to a normal distribution. For comparisons among 3 or more groups, assumptions (normality and homogeneity of variance) were checked by shapiro.test and leveneTest functions of ggpubr package in R. If assumptions were met, we proceeded with ANOVA followed by Tukey’s test if ANOVA was significant. If assumptions were violated, the kruskal.test() function was performed, with a post-hoc test conducted after a statistically significant Kruskal–Wallis result. Statistical significance was determined as a two-tailed *p* value of less than 0.05; *, *p* < 0.05; **, *p* < 0.01; ***, *p* < 0.001. All statistical analyses were conducted using R v4.3.1 including receiver operator characteristic (ROC) analyses and Kaplan–Meier (KM) curve.

## Results

### Baseline characteristics of cohorts

From April 2022 to September 2023, a discovery cohort consisting of 55 participants and a validation cohort of 151 participants were enrolled in this study. The demographic and clinicopathological characteristics for all participants can be found in Table 1. In discovery cohort, patients of sepsis group had a higher median age (69 years) than those from other groups (*p* = 0.002). In validation cohort, more male patients were found in sepsis group than in other groups (*p* = 0.03). Notably, the sepsis group demonstrated the highest level of illness severity. Patients from sepsis group exhibited both significantly higher Acute Physiology and Chronic Health Evaluation II (APACHE II) scores (*p* < 0.001) and SOFA scores (*p* < 0.001) than those from surgery group.

**Table 1:** Characteristics of the discovery cohort and validation cohort

Characteristics	Discovery cohort				Validation cohort		
	Healthy donor N=10	Mild infection N=15	Surgery N=15	Sepsis N=15	Mild infection N=49	Surgery N=36	Sepsis N=66
Age-- yr	51±7	51±20	57±12	69±10	66±14	62±12	67±12
Gender-- No. (%)							
Male	6(60)	10(67)	9(60)	9(60)	28(57)	17(47)	48(73)
Female	4(40)	5(33)	6(40)	6(40)	21(43)	19(53)	18(27)
Coexisting conditions -- No. (%)							
Diabetes		0	1(7)	6(40)	7(14)	4(11)	13(20)
Hypertension		1(7)	8(53)	4(27)	22(45)	18(50)	36(55)
Heart disease		0	15(100)	4(27)	8(16)	36(100)	16(24)
Chronic pulmonary disease		2(13)	0	1(7)	8(16)	1(3)	4(6)
Most common Primary sources of infection -- No. (%)							
Pneumonia		4(27)		4(27)	45(92)		31(47)
Urinary tract infection		2(13)		3(20)	3(6)		4(6)
Intra-abdominal infection		9(60)		8(53)	1(2)		20(30)
Bloodstream infection		0		0	0		11(17)
Laboratory Testing							
WBC count (IQR) -- 1000/mm3		12.51(8.38-18.10)	9.82(9.15-11.41)	14.48(4.73-19.50)	7.34(5.51-10.27)	10.51(8.45-12.33)	12.93(6.80-17.48)
CRP (IQR) -- mg/L		167.17(102.53-198.74)	39.04(30.41-64.25)	200.71(149.46-237.45)	39.85(24.23-107.00)	41.91(23.12-61.67)	146.48(67.49-228.72)
PCT (IQR) -- ng/mL		0.71(0.18-1.38)	0.76(0.42-3.30)	60.9(15.40-100.00)	0.12(0.06-0.35)	0.34(0.15-1.00)	8.07(1.64-68.68)
Creatinine (IQR) -- μmol/L		67(60-84)	94(64-119)	160(130-176)	60(51-71)	72(60-86)	107(80-181)
ALT (IQR) --U/L		20(16-31)	14(12-21)	33(15-53)	19(10-37)	16(11-22)	22(14-59)
AST (IQR) -- U/L		23(18-26)	26(23-58)	56(39-84)	22(15-38)	40(25-59)	46(28-80)
TBIL (IQR) -- μmol/L		11.8(8.9-17.5)	16.3(15.1-21.3)	28.5(21.8-45.8)	6.6(5.3-12.9)	14.2(10.9-19.4)	15.7(10.5-36.0)
APACHE II score			10.73±2.40	22.60±6.22		12.94±3.72	22.83±7.09
SOFA score		<2	4.47±2.00	10.53±2.67	<2	6.22±2.32	10.22±4.55

WBC: white blood cell; CRP: C-reactive protein; PCT: procalcitonin; ALT: alanine transaminase; AST: aspartate transaminase; APACHE II: Acute physiology and chronic health evaluation II; SOFA: Sequential Organ Failure Assessment

### Visualization of immune cell subsets

To elucidate the peripheral immune landscape, we conducted CyTOF analysis on the discovery cohort. All CyTOF data were subjected to gating procedures to identify viable single PBMC from patients’ blood samples. Subsequently, a total of 30 clusters were defined based on characteristic cell surface markers (Supplemental Fig. 1A, B). Several cell clusters showed discrepant proportions among healthy, mild infection, surgery and sepsis groups (Supplemental Fig. 1C).

**Fig. 2:**
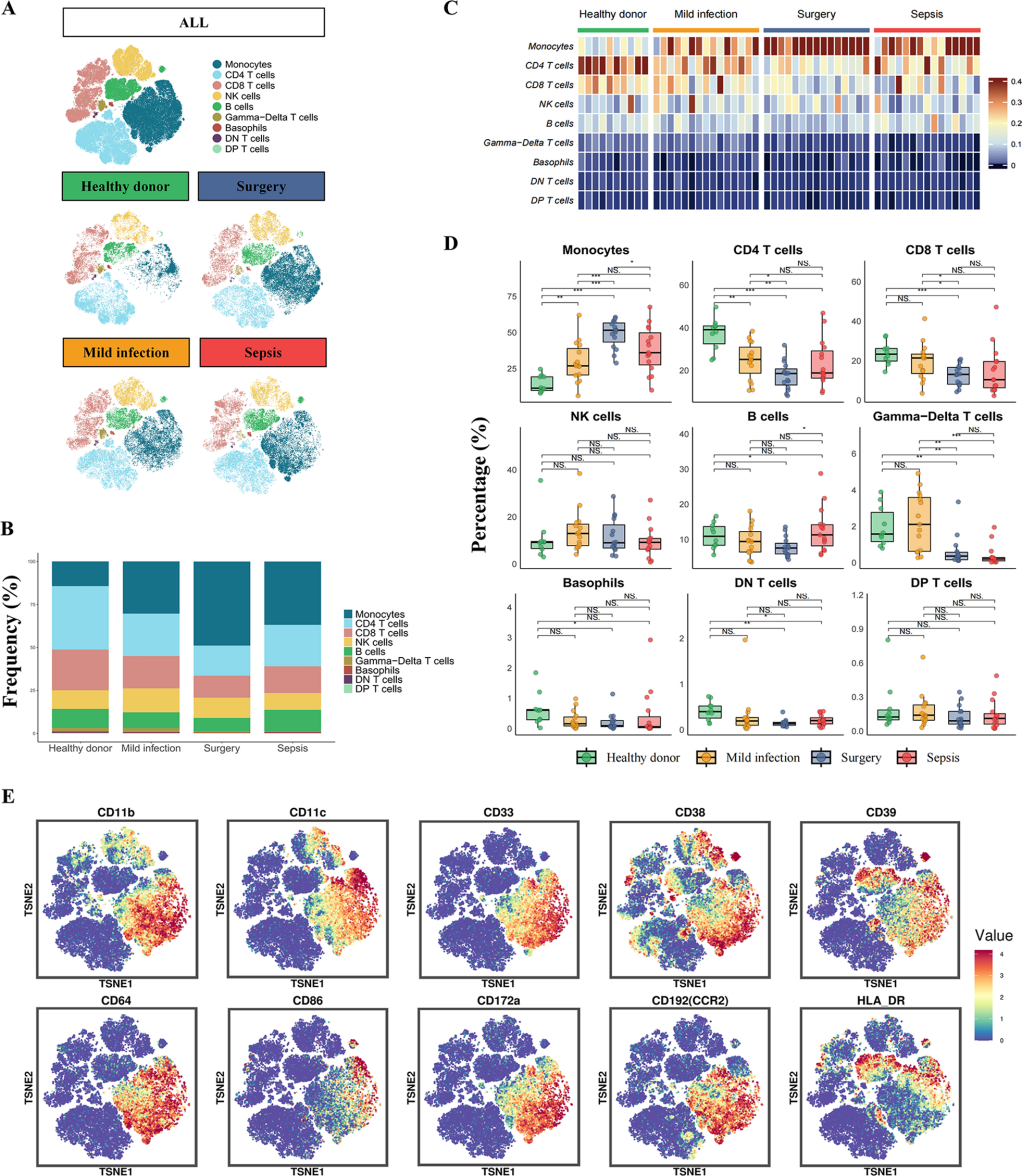
CyTOF analysis of peripheral blood mononuclear cells in discovery cohort. **A** t-SNE analysis showed a visualized map of major immune cell subsets in each group. **B** Changes in the composition of nine immune cell types among different groups. **C** Heatmap illustrated the alteration patterns of major immune cell subsets across different groups. **D** Box plots displayed the differential distributions of major immune cell subsets among four groups (* for *p* < 0.05, ** for *p* < 0.01, and *** for *p* < 0.001). **E** Differential expressions of ten immune-related cell markers on monocytes in the sepsis group. *CyTOF* mass cytometry time-of-flight. *t-SNE* t-distributed stochastic neighbor embedding. *NK cells* natural killer cells. *DN T cells* double negative T cells. *DP T cells* double positive T cells

The 30 clusters were then categorized into 9 distinct immune cell types: monocytes, CD4 T cells, CD8 T cells, natural killer (NK) cells, B cells, Gamma–Delta T cells, basophils, double negative (DN) T cells, and double positive (DP) T cells (Fig. 2A, and Supplemental Table 4). Monocytes, CD4 T cells, CD8 T cells, NK cells, and B cells constituted the major components of PBMCs in 4 groups (Fig. 2B), and the former 3 immune cell types presented the most dramatic alterations within different groups (Fig. 2C, D). Monocytes exhibited notably higher abundance in mild infection, surgery and sepsis groups compared with healthy donors, indicating the pivotal role of monocytes at the early stage of both infectious and non-infectious inflammation conditions (Fig. 2C).

The increase in monocytes and the decrease in T lymphocytes within 48 h of infection diagnosis or following cardiac surgery were consistent with the initiation of innate immunity during early response to inflammation. It’s worth mentioning that, the proportion of monocytes in blood from sepsis patients tended to be higher than from mild infection cases though without statistical significance (p = 0.05), suggesting that the functional changes rather than number alterations of monocytes might contribute to the development of sepsis. Therefore, we examined 42 markers of all cell clusters and identified 10 markers with distinct expressions in monocytes than other immune cells from all 4 groups (Supplemental Fig. 1D). Furthermore, the 10 cell markers also exhibited heterogeneous expression patterns within monocytes in sepsis group (Fig. 2E).

### Lower expression of monocytic CD39 in sepsis

**Fig. 3:**
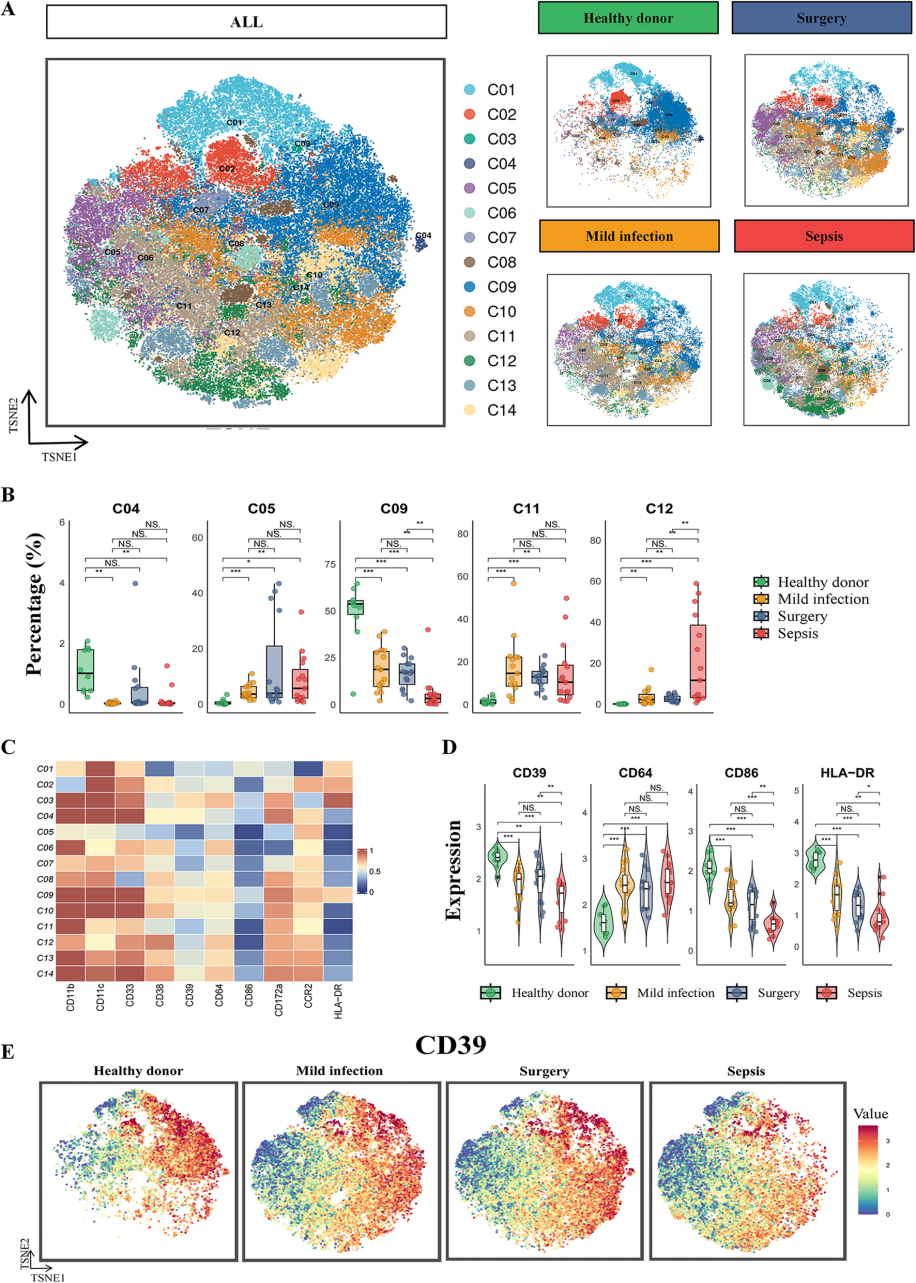
CyTOF analysis of monocytes in discovery cohort. **A** Monocytes from the total four groups were classified into 14 subpopulations based on immune-related cell markers, and these subpopulations showed distinct distributions across different groups. **B** Five of the 14 monocyte subsets displayed dramatic changes between healthy and inflammatory conditions. **C** Heatmap illustrated the expression of 10 immune-related cell markers across the 14 monocyte subpopulations. **D** CD39, CD64, CD86, and HLA–DR had differential expressions between the healthy group and the other three groups (* for *p* < 0.05, ** for *p* < 0.01, and *** for *p* < 0.001). **E** t-SNE plots demonstrated that CD39–low-expressed cells constituted the main population of monocytes in septic patients. *CyTOF* mass cytometry time-of-flight. *t-SNE* t-distributed stochastic neighbor embedding. *HLA–DR* human leukocyte antigen–DR

To further explore the variability of monocytes, we used these cell markers to conduct cell clustering analyses and classified monocytes from the total 4 groups into 14 subpopulations. The t-distributed stochastic neighborhood embedding (t-SNE) plots revealed the differential distributions of the 14 subpopulations between healthy and inflammation settings (Fig. 3A). Notably, monocytes from C04 and C09 populations showed a sharp decrease in mild infection and sepsis groups compared with healthy patients. Meanwhile, C05, C11 and C12 monocytes were dramatically increased in inflammation cases than in healthy donors (Fig. 3A, B and Supplemental Fig. 2). Furthermore, we compared the 5 subpopulations within the inflammation settings and we found that, sepsis patients had the lowest proportion of C09 monocytes and the highest proportion of C12 monocytes compared with mild infection and non-infectious inflammation (surgery) patients (Fig. 3B).

We also analyzed the expression of 10 cell markers in all 14 subpopulations of monocytes. Interestingly, C04 and C09 cells harbored relatively higher CD39 expression, while C05, C11 and C12 monocytes showed comparatively lower CD39 expression (Fig. 3C). Additionally, CD39, CD64, CD86, and human leukocyte antigen–DR (HLA–DR) exhibited differential expressions between the healthy group and the other three groups (Fig. 3D). Especially in sepsis patients, monocytes presented the lowest CD39, CD86 and HLA–DR expressions than mild infection and surgery patients. As validated in the t-SNE plots, CD39 high-expressed monocytes were dramatically reduced, while cells with low CD39 expression constituted the main subpopulation of monocytes in sepsis (Fig. 3A, E).

### Validation of monocytic CD39 expression in sepsis

**Fig. 4:**
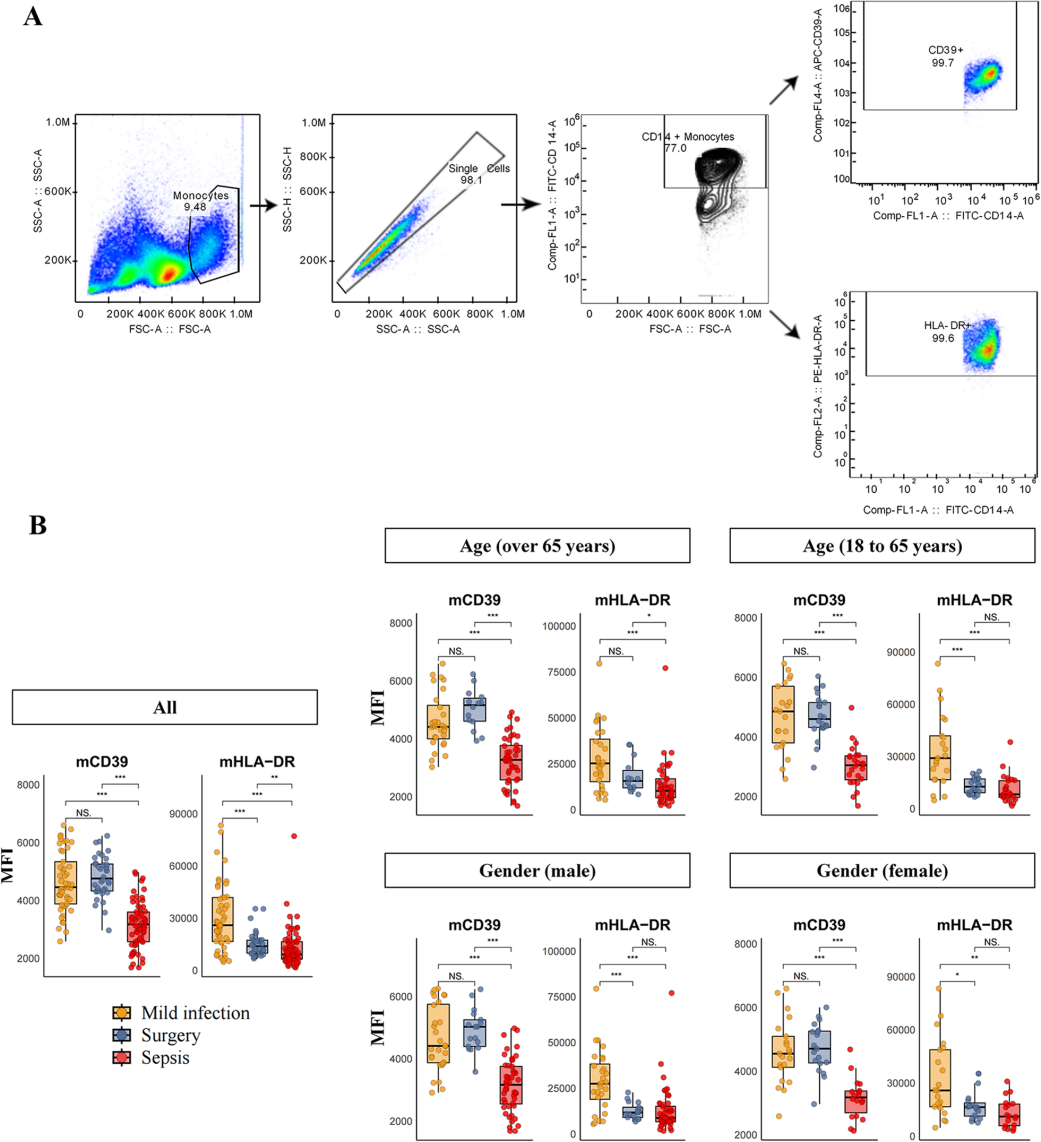
Flow cytometry of monocytes in validation cohort. **A** Gating strategy of fluorescent flow cytometry for monocytes in the validation cohort. **B** Validation of mCD39 and mHLA–DR expressions among mild infection, surgery and sepsis groups, and stratified analyses based on age and gender (* for *p* < 0.05, ** for *p* < 0.01, and *** for *p* < 0.001). *mCD39* monocytic CD39. *mHLA–DR* monocytic human leukocyte antigen–DR. *MFI* median fluorescence intensity

Although there were reports about the expression of CD39 in various diseases [19, 20], no existing studies have focused on the role of monocytic CD39 (mCD39) in sepsis. To ease the future application of CD39 in clinical practice, we employed the cell flow cytometry technique in 3 validation groups to verify our findings of mCD39, as well as monocytic HLA–DR (mHLA–DR) whose role in sepsis was studied in previous reports [21–23]. The gating strategy is depicted in Fig. 4A. By comparing the mean fluorescence intensity (MFI) values, we found that the expression levels of mCD39 and mHLA–DR in the sepsis group were significantly lower compared to those in the mild infection and surgery groups (Fig. 4B). This finding was consistent with the conclusion drawn from the discovery cohort. As discovered in Table 1, patients’ gender and age differed among our study groups. Therefore, subgroup analyses based on age and gender were performed, resulting in similarly significant differences in mCD39 expressions between the sepsis group and the other 2 groups including mild infection and surgery patients (Fig. 4B). However, no significant differences were detected in mHLA–DR expression levels between the sepsis and surgery groups in the stratified analyses in 18–65 years, male, and female populations (Fig. 4B).

### mCD39 in predicting diagnosis and prognosis of sepsis

**Fig. 5:**
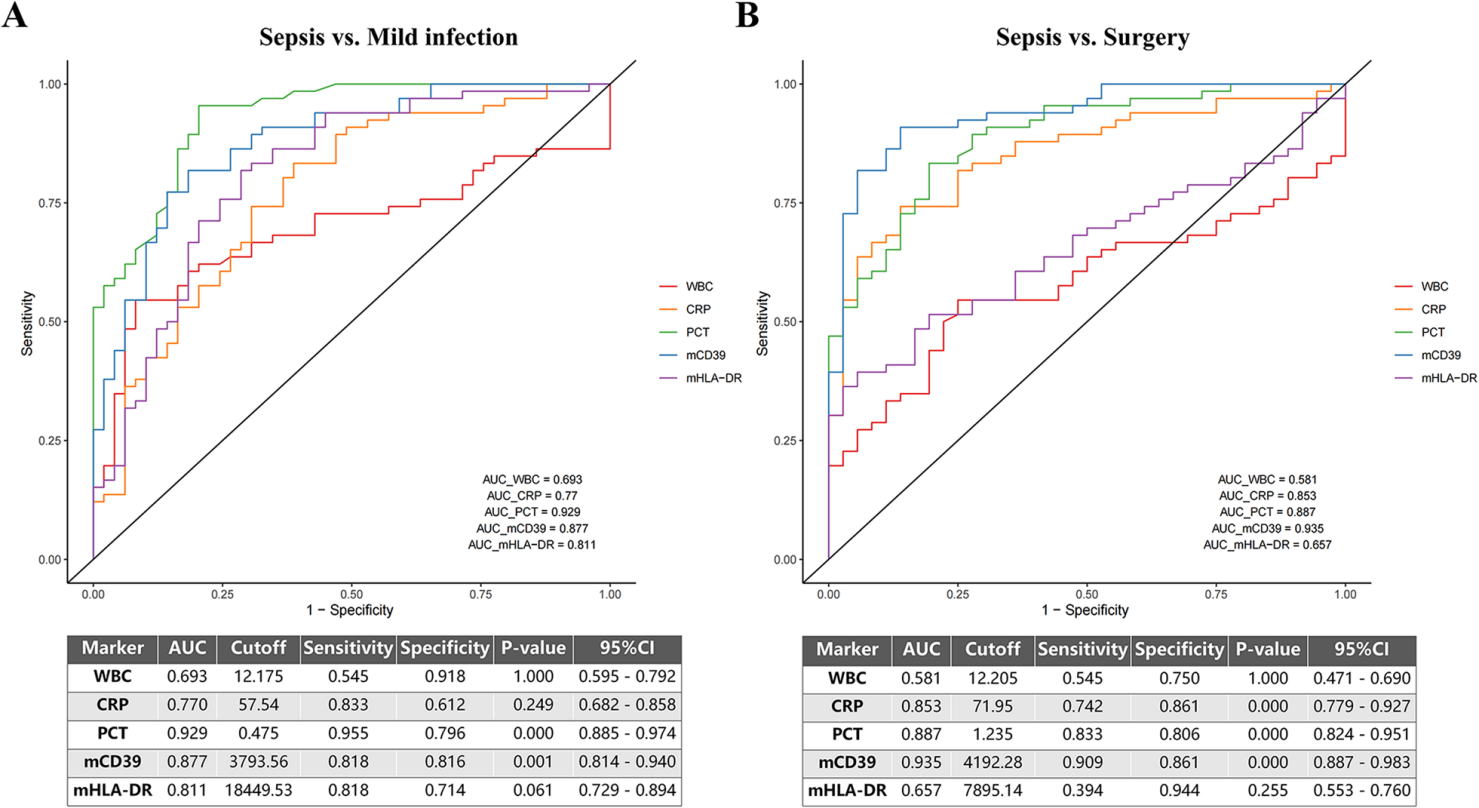
ROC curves displaying the capability of CD39 and commonly used biomarkers in sepsis diagnosis. **A** PCT and mCD39 exhibited superior ability in distinguishing sepsis and mild infection. **B** mCD39 showed the highest AUC for the differentiation of sepsis from non-infectious inflammation (surgery group). ROC: receiver operating characteristic. WBC: white blood cell count. *CRP* C-reactive protein. *PCT* procalcitonin. *mCD39* monocytic CD39. *mHLA–DR* monocytic human leukocyte antigen–DR. *AUC* area under the curve. *CI* confidence interval

To further explore the clinical applications of mCD39, we compared patients' mCD39 with mHLA–DR, WBC, CRP, and PCT as diagnostic indicators to assess its predictive value in sepsis. ROC curves demonstrated that PCT and mCD39 had significantly stronger power to distinguish sepsis patients from mild infection patients (Fig. 5A). MFI of mCD39 lower than 3793.56 in flow cytometry was a possible predictor for the early diagnosis of sepsis, with the area under the curve (AUC) of 0.877 (*p* = 0.001). The well-known inflammation marker PCT also displayed a high AUC of 0.929 (*p* < 0.001). In the settings between sepsis and non-infectious inflammation, mCD39 showed the ability for sepsis diagnosis, with an AUC of 0.935 and a *p* value < 0.001 (Fig. 5B). In addition, in subgroups stratified by age and gender, mCD39 consistently showed capable predictive value (Supplemental Fig. 3).

**Fig. 6:**
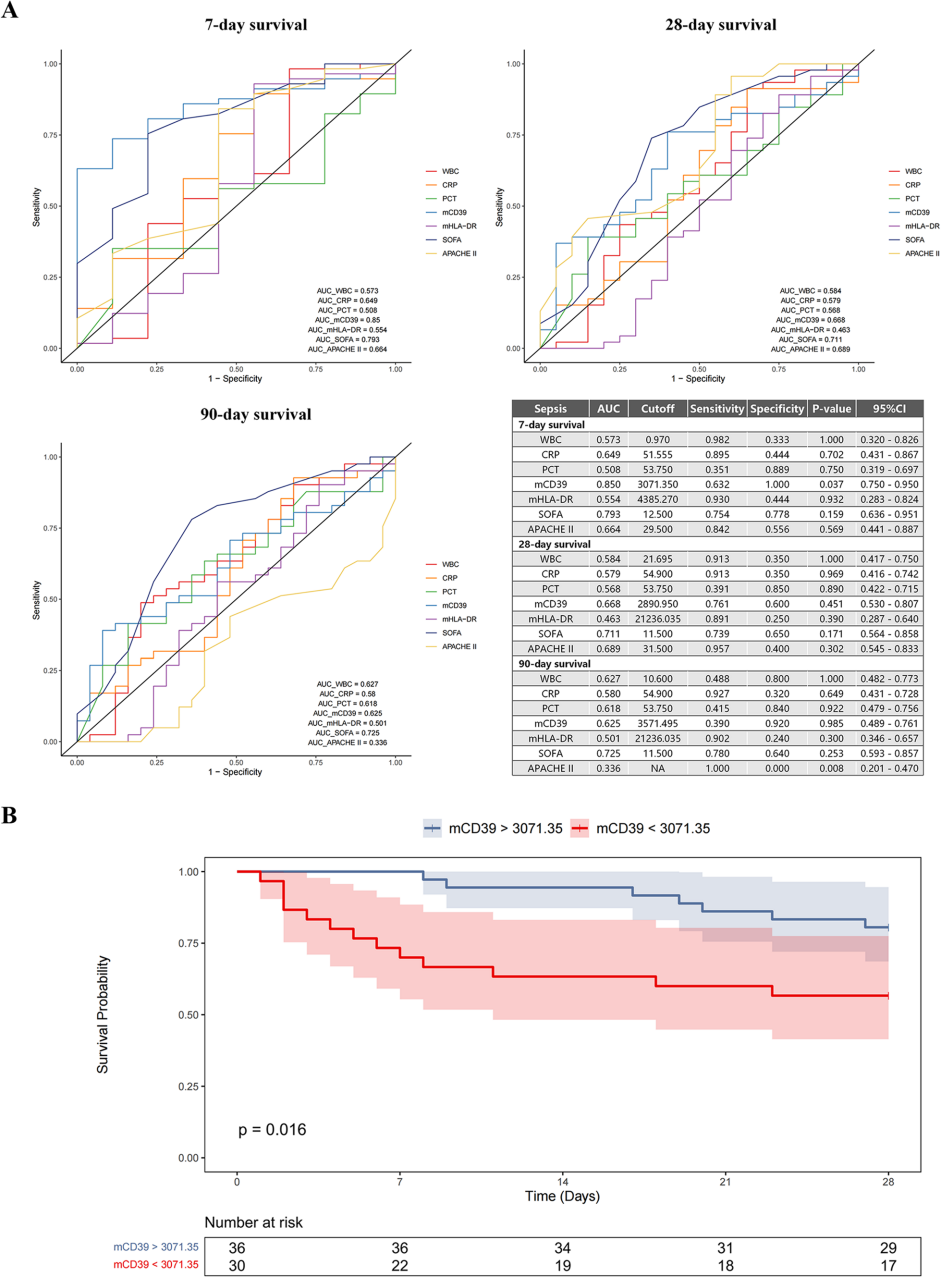
Role of mCD39 in predicting the prognosis of sepsis. **A** ROC curves examined the capability of WBC, CRP, PCT, mCD39, mHLA–DR, SOFA and APACHE II for the prediction of short-term and long-term prognosis in sepsis patients. **B** Kaplan–Meier curves compared the prognosis of sepsis patients with different mCD39 expressions. *ROC* receiver operating characteristic. *WBC* white blood cell count. *CRP* C-reactive protein. *PCT* procalcitonin. *mCD39* monocytic CD39. *mHLA–DR* monocytic human leukocyte antigen–DR. *SOFA* Sequential Organ Failure Assessment. *Apache II* Acute Physiology and Chronic Health Evaluation II. *AUC* area under the curve. *CI* confidence interval

In addition, we generated the ROC curves to predict the prognosis of sepsis patients. The endpoints were defined as the overall survival at 7 days, 28 days, and 90 days after diagnosis of sepsis (Fig. 6A). Among the seven indicators (WBC, CRP, PCT, mCD39, mHLA–DR, SOFA and APACHE II), only mCD39 exhibited strong capability in predicting the 7-day survival with a cutoff value of MFI lower than 3071.35 (AUC = 0.85, *p* = 0.037). Subsequently, sepsis patients were divided into two groups based on the cutoff value of mCD39. The Kaplan–Meier curve in Fig. 6B demonstrated that patients with mCD39 MFI lower than 3071.35 had significantly worse survival than patients with higher mCD39 expressions (28-day overall survival rates were 56.7% vs. 80.6%, *p* = 0.016). Furthermore, lower expression of mCD39 trended to correlate with higher SOFA score (Supplemental Fig. 4). However, the *p* value showed no statistical significance (*p* = 0.077).

## Discussion

Although the Surviving Sepsis Campaign approved the significance of biomarkers in sepsis, the revised guidelines in 2021 did not provide any recommendations regarding the utilization of biomarkers for sepsis diagnosis or prognosis [24]. Using CyTOF and flow cytometry techniques in the present study, we identified and validated a novel biomarker mCD39, which was significantly decreased in monocytes from sepsis patients than those from mild infection and non-infectious inflammation patients. Furthermore, when compared with the conventional immune indicator (mHLA–DR), the classic inflammatory indicators (WBC, CRP and PCT) and the classic scoring systems (SOFA and APACHE II), low expression of mCD39 exhibited potential ability in diagnosis of sepsis, as well as powerful capability of short-term prognosis prediction at the early stage of sepsis.

In the discovery cohort, we detected significant increase of monocytes, as well as the decrease of CD4 T cells and CD8 T cells, in blood samples obtained within 48 h from both infectious and non-infectious inflammation patients. Monocytes are the major components of innate immunity system that could rapidly respond to inflammation at an early stage before acquired immunity is initiated. T lymphocytes represent critical roles in acquired immunity. The alterations of monocyte and T lymphocyte proportions in this study were consistent with changes of immune response patterns during different stages of inflammation. Previous research has shown that exhaustion of CD4 T cells and CD8 T cells indicates poor prognosis in sepsis patients [25, 26]. Although no statistical difference was found in the proportions of monocytes between mild infection and sepsis groups, multiple cell markers in monocytes displayed discrepant expressions between 2 groups, implying that functional change of monocytes might play a pivotal role in sepsis.

In the CyTOF analyses, monocytes exhibited notably lower CD39, CD86 and HLA–DR expressions in sepsis patients than in mild infection patients. Furthermore, while CD39 high-expressed monocytes were sharply reduced, cells with low CD39 expression became the main subpopulation of monocytes in sepsis. HLA–DR in monocytes has been identified as a crucial immune indicator for sepsis [21–23], while CD86 has also been examined in sepsis research [27, 28]. No existing studies have explored the effect of mCD39 in sepsis.

This study demonstrated that mCD39 had better capability for the diagnosis of sepsis in comparison with mHLA–DR, which was widely recognized as an important marker reflecting the immune status of sepsis. The reduced expression of mHLA–DR has been related to the development and outcome of sepsis [29–32]. PCT, another important marker of inflammation and infection, also exhibited high accuracy for sepsis diagnosis in previous research findings [33, 34]. This study revealed that the diagnostic ability of mCD39 for sepsis was comparable to that of PCT. However, the relatively low specificity of PCT hampers its ability in distinguishing non-infectious inflammation or trauma, and infectious disease.

It is noteworthy that the AUC value of mCD39 reached 0.85 (*p* < 0.05) specifically for 7-day mortality, as compared to PCT (with an AUC of 0.508), SOFA score (AUC = 0.793) and APACHE II score (AUC = 0.664). Both SOFA and APACHE II are known as strong prognostic predictors for sepsis patients [35, 36]. There was a negative correlation trend between SOFA and mCD39. However, no statistical significance was obtained (*p* = 0.077). We speculated that mCD39 expression may partially reflect the severity of sepsis, but it might act with other factors in the progression of sepsis. The sample size might also limit the statistical analysis. Our finding suggested a potential association between mCD39 and the immune status at the early stage of sepsis, during which sepsis triggers an excessive activation of innate immunity in the acute phase. In addition, the excessive inflammatory response has been demonstrated to contribute to premature mortality in individuals diagnosed with sepsis [37, 38]. Our results indicated that low expressions of mCD39 could act as a powerful predictor of poor prognosis in sepsis patients.

Currently, there haven't been any published research on mCD39 in sepsis. CD39 refers to a transmembrane Ectonucleoside triphosphate diphosphohydrolase-1 (ENTPD-1), which possesses two transmembrane domains and one extracellular domain. In terms of pathogenesis, cells that experience stress or injury, such as infection or trauma, could release adenosine triphosphate (ATP) into the extracellular environment, thereby facilitating pro-inflammatory reactions [39]. CD39 plays a crucial role in this process by hydrolyzing extracellular ATP (eATP) into adenosine monophosphate (AMP), while CD73 converts AMP into the immunosuppressive nucleoside adenosine (ADO). CD39 is considered a rate-limiting enzyme in this cascade, serving as an immunological switch that transitions the ATP-mediated pro-inflammatory environment to an ADO-mediated anti-inflammatory state, so as to enhance immune equilibrium and alleviate organ impairment resulting from excessive pro-inflammatory reactions [40].

The expression of CD39 can be observed in diverse immune cells. Savio et al. discovered that the presence of CD39 in macrophages restricted the pro-inflammatory signaling of ATP–P2X7 receptors, and their study on septic CD39-/- mice demonstrated an elevated level of inflammatory cytokines compared to the control group of wild-type mice [20]. This fundamental investigation provides some elucidation on the involvement of CD39 in sepsis and makes our research results more convincing. In another study, Nascimento et al. demonstrated that septic individuals displayed an expansion of CD39^hi^ plasmablasts and an accumulation of adenosine, which are considered significant factors contributing to sepsis-induced immunosuppression [19]. It is noteworthy to highlight that our research diverges from theirs in several crucial aspects. First, while they specifically focused on investigating the expression of CD39 on plasmablasts, this study examined its expression on monocytes. In addition, the timing of blood sample collection differed between the two studies, with Nascimento et al. collecting samples at the 7-day mark of sepsis, suggesting that the individuals included in their study were at varying stages of the disease compared to the blood samples we obtained.

This study is subject to several limitations. First, it was conducted exclusively at a single center, which may limit the universality of the findings. Second, the observational design and relatively small sample size also potentially impact the statistical power and precision of the results. Third, the methods section described that the blood samples were collected within 48 h. In our pipeline, the time of blood sample collection was 6 am in the next day and was usually within 24 h of diagnosis/following cardiac surgery. However, there were a few cases in which patients were transferred to our department at midnight, and the blood sample collection the next day would be more than 24 h later. Based on the advocation of Surviving sepsis campaign guidelines, we practiced the early administration of antibiotics, which would possibly affect our results. In addition, the CyTOF we used as a massive screening for immune markers was comparatively expensive and time-consuming. Therefore, in the validation part, we chose the flow cytometry method which was easier and more feasible. However, an improved technique is still needed to enhance the efficacy of mCD39 test and to facilitate wide clinical applications. Further experimental researches are needed to explore effects and mechanisms of CD39 in the functional changes of monocytes in sepsis.

## Conclusions

In this study, we discovered that monocytes with lower CD39 expression exhibited high abundance in sepsis patients. Low expression of CD39 on monocytes was a potential diagnostic biomarker for sepsis, and was a powerful predictor of poor prognosis in sepsis patients.

## Supplementary Information

Supplementary Material 1.

## Data Availability

The data that support the findings of this study are not openly available due to reasons of sensitivity and are available from the corresponding author upon reasonable request.

## Abbreviations

SIRS: Systemic inflammatory response syndrome
SOFA: Sequential organ failure assessment
WBC: White blood cell count
CRP: C-reactive protein
PCT: Procalcitonin
CyTOF: Mass cytometry time-of-flight
FACS: Fluorescence-activated cell sorting
PBMCs: Peripheral blood mononuclear cells
mCD39: Monocytic CD39
HIV: Human immunodeficiency virus
EDTA: Ethylenediaminetetraacetic acid
PBS: Phosphate buffered saline
BSA: Bovine serum albumin
FBS: Fetal bovine serum
DMSO: Dimethyl sulfoxide
DNA: Deoxyribonucleic acid
t-SNE: T-Distributed stochastic neighborhood embedding
Apache II: Acute Physiology and Chronic Health Evaluation II
ANOVA: Analysis of variance
ROC: Receiver operator characteristic
KM: Kaplan–Meier
NK: Nature killer
DN: Double negative
DP: Double positive
HLA–DR: Human leukocyte antigen–DR
mHLA–DR: Monocytic human leukocyte antigen–DR
MFI: Mean fluorescence intensity
AUC: Area under the curve
ENTPD-1: Ectonucleoside triphosphate diphosphohydrolase-1
ATP: Adenosine triphosphate
eATP: Extracellular ATP
AMP: Adenosine monophosphate
ADO: Adenosine
CI: Confidence interval
ALT: Alanine transaminase
AST: Aspartate transaminase
