# Supplementary material for: Low expression of CD39 on monocytes predicts poor survival in sepsis patients
Source: J Intensive Care. 2025 Mar 10;13:12. doi: 10.1186/s40560-025-00784-0 (PMC11892179; doi:10.1186/s40560-025-00784-0)
Supplement: Supplementary file 1 — Supplementary Material 1. [file 40560_2025_784_MOESM1_ESM.docx]

# Low Expression of CD39 on Monocytes Predicts Poor Survival in Sepsis Patients

Hangyang Li, Peili Ding, Yuyu Nan, Zhenping Wu, Ning Hua, Lixi Luo, Qinghua Ji, Fangfang Huang, Guobin Wang, Hongliu Cai, Saiping Jiang, Wenqiao Yu

**Supplementary Materials**

**Supplementary Methods:**

**Cell isolation, cryopreservation and recovery**

Peripheral blood samples were collected from each participant (5mL per person). Peripheral Blood Mononuclear Cells (PBMCs) were isolated from whole blood using Ficoll density gradient centrifugation with Ethylenediaminetetraacetic Acid (EDTA) as an anticoagulant. Following the isolation, the cell pellet was washed with fluorescence-activated cell sorting (FACS) buffer (1×phosphate buffered saline (PBS) supplemented with 0.5% bovine serum albumin (BSA)), and the supernatant was discarded before resuspending the cells for counting. Quality control for blood samples included a requirement of at least 3×10^6^ viable cells with a minimum viability of 85%. Once isolated and counted, the cells were resuspended in a pre-cooled cryopreservation solution (10% Fetal Bovine Serum (FBS) + 90% Dimethyl Sulfoxide (DMSO)) at a density of 1~5×10^6^/mL. Subsequently, the cells were gently mixed and aliquoted into cryovials. These cryovials were then placed in a programmable cooling box and stored at -80℃ for at least 4 hours and transferred to liquid nitrogen for long-term storage.

To recover the cells, the cryovials were thawed in a 37°C water bath for 2-3 minutes. Once fully thawed, the cells were carefully transferred to a 15mL centrifuge tube within a biosafety cabinet. The cryovial was washed with 1mL of complete culture medium, and the remaining medium was transferred to the 15mL centrifuge tube. The volume was adjusted to 5mL, and the conical tube was gently swirled. Subsequently, the cells were centrifuged at 400g at room temperature, washed, and resuspended using FACS. Cell counting and viability assessment were performed using Trypan Blue. Reagent information is shown in Supplemental Table 1.

**CyTOF staining and data acquisition**

The mass cytometry time-of-flight (CyTOF) antibody panel in this study is shown in the Supplemental Table 2. Each sample was meticulously processed to collect a maximum of 3×10^6^ cells, and cisplatin staining was employed to selectively exclude dead cells. After the FACS washing process, the cells were subjected to incubation with Fc receptor blocking solution with a cocktail of surface antibodies on ice for a duration of 30 minutes. Subsequently, a second round of FACS washing was conducted before fixing the cell membranes and staining intracellular antibody indicators. An additional FACS washing step was performed, followed by overnight fixation in a DeoxyriboNucleic Acid (DNA) staining solution prepared using Fix and Perm buffer. Following an additional FACS washing step, the cells were suspended in deionized water, thereby preparing them for analysis on the CyTOF system (Helios, Fluidigm). In order to guarantee precise instrument functionality, the CyTOF system was activated and the sampling line was installed. The instrument underwent self-testing and calibration procedures using Tuning solution and EQ beads. The sample cells were then resuspended in deionized water containing 10% EQ beads, and data acquisition was facilitated by employing a 40μm cell sieve for filtration.

**CyTOF analysis**

After a rigorous de-barcoding and standardization process, each sample was subjected to data cleaning using FlowJo V10 software, including the manual exclusion of debris and dead cells, thereby ensuring that only viable single immune cells remained. To decipher the underlying cellular heterogeneity, all immune cells were comprehensively analyzed using an unsupervised clustering algorithm in R v4.3.1. The resulting heatmap revealed distinct marker expression patterns, enabling precise annotation of cell subpopulations.

To perform intuitive visualization, a t-distributed stochastic neighborhood embedding (t-SNE) data dimensionality reduction algorithm was utilized. Following this visualization, rigorous statistical analysis was performed, aiming to distinctively classify the different cell subgroups. The results revealed that the monocyte subpopulation exhibited the most substantial variances.

Therefore, using FlowJo V10 software, monocytes were accurately gated, and the same clustering and analysis methods were applied to reveal the differences among clusters and markers within the monocyte population.

**Flow cytometry**

A three-marker panel (Supplemental Table 3) was designed for conventional fluorescent flow cytometry. Each sample was processed with 1×10^6^ cells collected individually. Fc receptor blocking was performed on ice for a duration of 20 minutes as part of the initial preparation to minimize non-specific binding and ensure the accuracy of subsequent analyses. Adhering to the manufacturer's instructions, a carefully prepared antibody mix, consisting of specific targets and fluorochromes, was incubated with the cells on ice for 30 minutes. This step allowed for accurate identification and characterization of cell populations based on surface markers. The cells were extensively washed with FACS buffer to remove any unbound antibodies and ensure a clean sample for analysis. The analysis was performed using the flow cytometer (Mindray), with data acquisition capturing a total of 200,000 events from the PBMCs. The focus of the analysis was on CD14 positive cells which was a common marker for monocytes, and a predefined gating logic was applied to ensure accurate identification and quantification of this cell population. This gating strategy was chosen based on its relevance to the research objectives and was applied consistently across all samples.

**Data collection**

Patients' clinical data were systematically gathered on the initial day (day 1), encompassing vital status, components of the Acute Physiology and Chronic Health Evaluation II (APACHE II) and Sequential Organ Failure Assessment (SOFA) score, as well as various clinical and biological parameters, including blood counts. Subsequently, vital status assessments were conducted on days 7, 28, and 90. Comprehensive baseline characteristics such as demographics, clinical profiles, laboratory findings, treatment modalities, and patient outcomes were meticulously extracted from electronic medical records.

**Supplementary tables:**

**Supplemental Table 1** Reagent information

| Reagent | Source | Catalog |
| --- | --- | --- |
| Maxpar® Antibody Labeling Kit | FLUIDIGM | 201300 |
| Percoll | GE Healthcare | 17-0891-09 |
| Ficoll-Paque PLUS | GE Healthcare | 17-1440-03 |
| MACS® Tissue Storage Solution | 130-100-008 | Miltenyi |
| Tumor Dissociation Kit, human | 130-095-929 | Miltenyi |
| gentleMACS™ C Tubes | 130-093-237 | Miltenyi |
| Phosphate-Buffered Saline | GENOM | GNM20012 |
| Bovine Serum Albumin | Sigma-Aldrich | V900933 |
| eBioscience™ Permeabilization Buffer (10X) | Thermo Fisher | 00-8333-56 |
| eBioscience™ Fixation/Permeabilization Concentrate | Thermo Fisher | 00-5123-43 |
| eBioscience™ Fixation/Permeabilization Diluent | Thermo Fisher | 00-5223-56 |
| Maxpar® Fix and Perm Buffer | FLUIDIGM | 201067 |
| Cell-ID™ Cisplatin-194Pt | FLUIDIGM | 201194 |
| Cell-ID™ Intercalator-Ir | FLUIDIGM | 201192B |
| Tuning Solution | FLUIDIGM | 201072 |
| EQ™ Four Element Calibration Beads | FLUIDIGM | 201078 |
| Washing Solution | FLUIDIGM | 201070 |

**Supplemental Table 2** CyTOF antibody panel

| Label | Marker | Clone | Source | Catalog | Staining |
| --- | --- | --- | --- | --- | --- |
| 89Y | CD45 | HI30 | BioLegend | 304002 | Surface |
| 115ln | CD3 | UCHT1 | BioXcell | BE0231 | Surface |
| 141Pr | CD56 | NCAM16.2 | BD | 559043 | Surface |
| 142Nd | TCRgd^*^ | 5A6.E9 | Homemade | NA | Surface |
| 142Nd | CD19^*^ | HIB19 | BioLegend | 333802 | Surface |
| 143Nd | CD27 | O323 | BioLegend | 302802 | Surface |
| 144Nd | CD14 | M5E2 | BioLegend | 301810 | Surface |
| 145Nd | IgD | IA6-2 | BioLegend | 348202 | Surface |
| 146Nd | CD123 | 6H6 | BioLegend | 306002 | Surface |
| 147Sm | IL-4 | MP4-25D2 | BioLegend | 500802 | Intracell |
| 148Nd | CD64 | 10.1 | BioLegend | 305016 | Surface |
| 149Sm | CD25 | 24212 | RD | MAB1020 | Surface |
| 150Nd | CD192(CCR2) | K036C2 | BioLegend | 357202 | Surface |
| 151Eu | CD172a | SE5A5 | BioLegend | 323802 | Surface |
| 152Sm | CD39 | A1 | BioLegend | 328202 | Surface |
| 153Eu | CD57 | HCD57 | BioLegend | 322325 | Surface |
| 154Sm | Ki-67 | SolA15 | Thermo | 14-5698-82 | Intracell |
| 155Gd | CD45RA | HI100 | BioLegend | 304102 | Surface |
| 156Gd | CD86 | Fun-1 | BD | 555655 | Surface |
| 157Gd | CD28 | CD28.2 | BioXcell | BE0291 | Surface |
| 158Gd | IFNγ | B27 | BioLegend | 506502 | Intracell |
| 159Tb | CD11c | BU15 | BioLegend | 337202 | Surface |
| 160Gd | CD33 | WM53 | BioLegend | 303419 | Surface |
| 161Dy | CX3CR1 | K0124E1 | BioLegend | 355702 | Surface |
| 162Dy | Foxp3 | PCH101 | Thermo | 14-4776-82 | Intracell |
| 163Dy | CD127 | A019D5 | BioLegend | 351302 | Surface |
| 164Dy | CD185(CXCR5) | RF8B2 | BD | 552032 | Surface |
| 165Ho | CD66b | G10F5 | BioLegend | 305102 | Surface |
| 166Er | CD69 | FN50 | BioLegend | 310902 | Surface |
| 167Er | CD197(CCR7) | G043H7 | BioLegend | 353222 | Surface |
| 168Er | TREM2 | 237920 | RD | MAB17291 | Surface |
| 169Tm | CD45RO | UCHL1 | BioLegend | 304202 | Surface |
| 170Er | T-bet | 4B10 | BioLegend | 644802 | Intracell |
| 171Yb | CD279(PD1) | EH12.2H7 | BioLegend | 329926 | Surface |
| 172Yb | CD38 | HIT2 | BioLegend | 303502 | Surface |
| 173Yb | IL-17A | BL168 | BioLegend | 512302 | Intracell |
| 174Yb | CD20 | 2H7 | BioLegend | 302302 | Surface |
| 175Lu | CD16 | 3G8 | BioLegend | 302014 | Surface |
| 176Yb | HLA-DR | L243 | BioLegend | 307612 | Surface |
| 197Au | CD4 | RPA-T4 | BioLegend | 300516 | Surface |
| 198Pt | CD8a | RPA-T8 | BioLegend | 301018 | Surface |
| 209Bi | CD11b | M1/70 | BioLegend | 101202 | Surface |

^*^CD19 and TCRgd channels are the same. They require co-expression with CD3 and either Gamma-Delta T cells or B cells.

**Supplemental Table 3** Flow cytometry panel

| Label | Marker | Clone | Source | Catalog | Staining |
| --- | --- | --- | --- | --- | --- |
| PE | HLA-DR | L243 | BioLegend | 307606 | Surface |
| APC | CD39 | A1 | BioLegend | 328210 | Surface |
| FITC | CD14 | M5E2 | BioLegend | 301804 | Surface |

**Supplemental Table 4** The subtype of immune cells within the CD45^+^ cell category

| Cluster | Cell type | Subtype |
| --- | --- | --- |
| C01 | CD4 T cells | Effector Memory T |
| C02 | CD4 T cells | Naïve T |
| C03 | CD4 T cells | Treg |
| C04 | CD4 T cells | Effector Memory T |
| C05 | CD4 T cells | Treg |
| C06 | CD4 T cells | Effector Memory T |
| C07 | CD4 T cells | Central Memory T |
| C08 | CD8 T cells | Naïve T |
| C09 | CD8 T cells | Effector Memory T |
| C10 | CD8 T cells | Effector T |
| C11 | CD8 T cells | Effector T |
| C12 | CD8 T cells | Effector Memory T |
| C13 | CD8 T cells | Naïve T |
| C14 | CD8 T cells | Effector Memory T |
| C15 | Gamma-Delta T cells |  |
| C16 | DN T cells |  |
| C17 | DP T cells |  |
| C18 | B cells | Naïve B |
| C19 | B cells | Switched Memory |
| C20 | B cells | Plasma |
| C21 | NK cells | Cytotoxic NK |
| C22 | NK cells | Cytotoxic NK |
| C23 | Monocytes | Non-Classical |
| C24 | Monocytes | Classical |
| C25 | Monocytes | Classical |
| C26 | Monocytes | Classical |
| C27 | Monocytes | Classical |
| C28 | Monocytes | Classical |
| C29 | Monocytes | Classical |
| C30 | Basophils |  |

**Supplementary figures:**

**
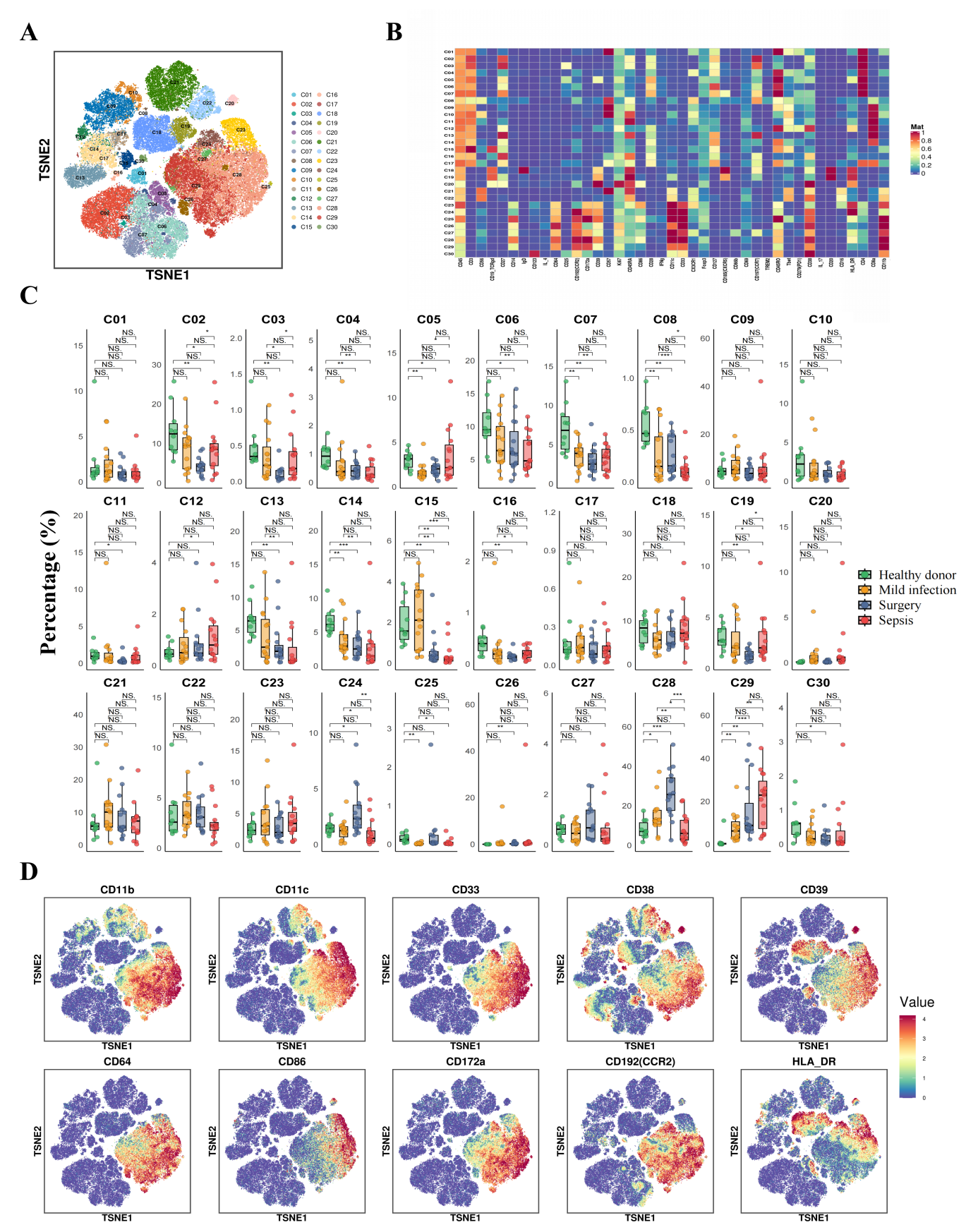
**

**Supplemental Fig. 1 CyTOF analysis of peripheral blood mononuclear cells. A** The t-SNE maps were utilized to visually represent the PBMCs that were analyzed with distinct immune cell subsets across various groups. The analysis encompassed a total of 30 clusters within the immune cell population. **B** A heatmap was constructed to illustrate the normalized expressions of markers present in the peripheral immune cells. Types of various immune cell subsets were presented in Supplemental Table 4. **C** The distribution of immune cell subsets among the different groups was graphically presented using box plots (* for p < 0.05, ** for p < 0.01, and *** for p < 0.001). **D** The t-SNE heatmap provided a visual representation of cell markers that exhibit high expression levels on monocytes.

CyTOF: mass cytometry time-of-flight. t-SNE: t-distributed stochastic neighbor embedding. PBMCs: peripheral blood mononuclear cells.

**
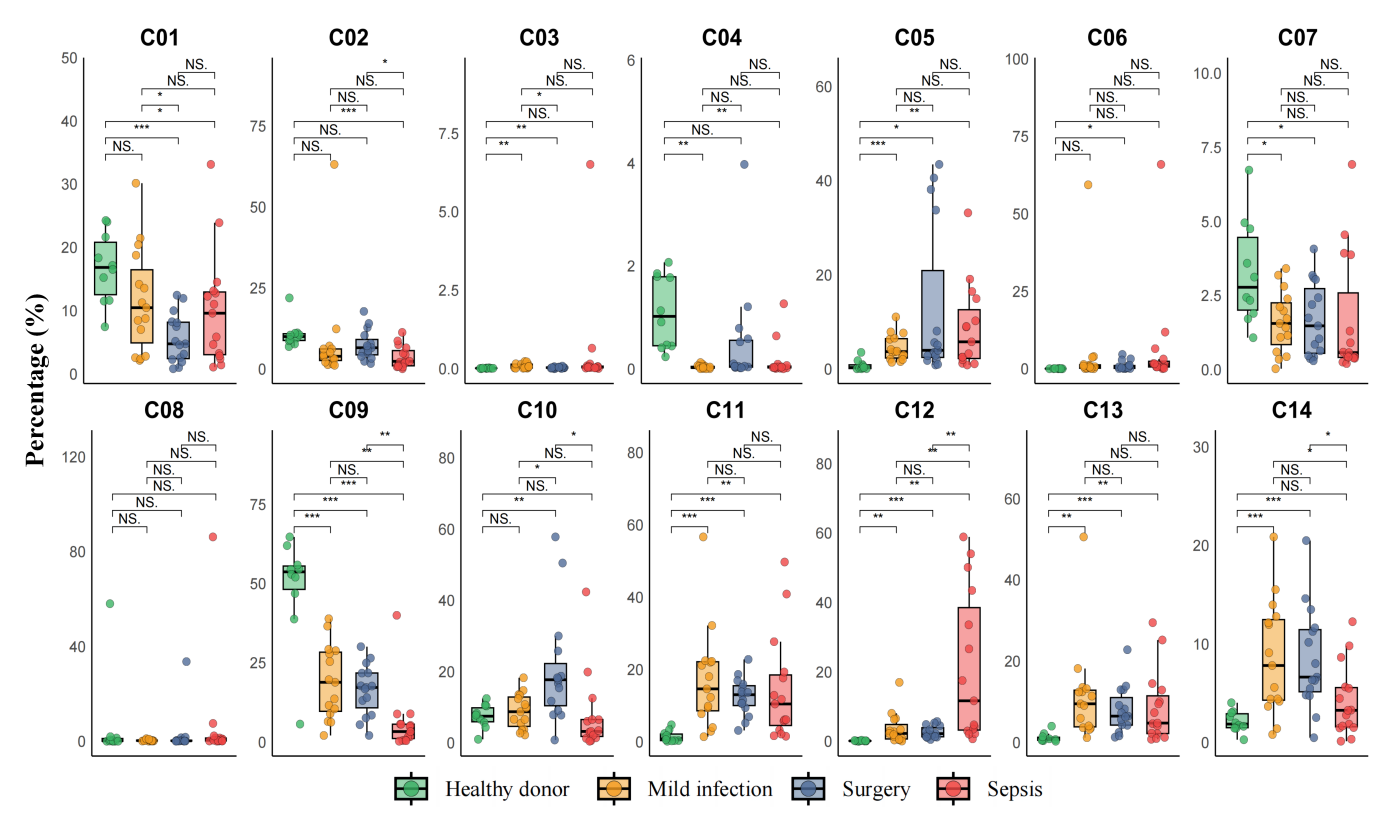
 Supplemental Fig. 2** The distribution of monocyte subsets among the different groups was visually represented through box plots (* for p < 0.05, ** for p < 0.01, and *** for p < 0.001).

**
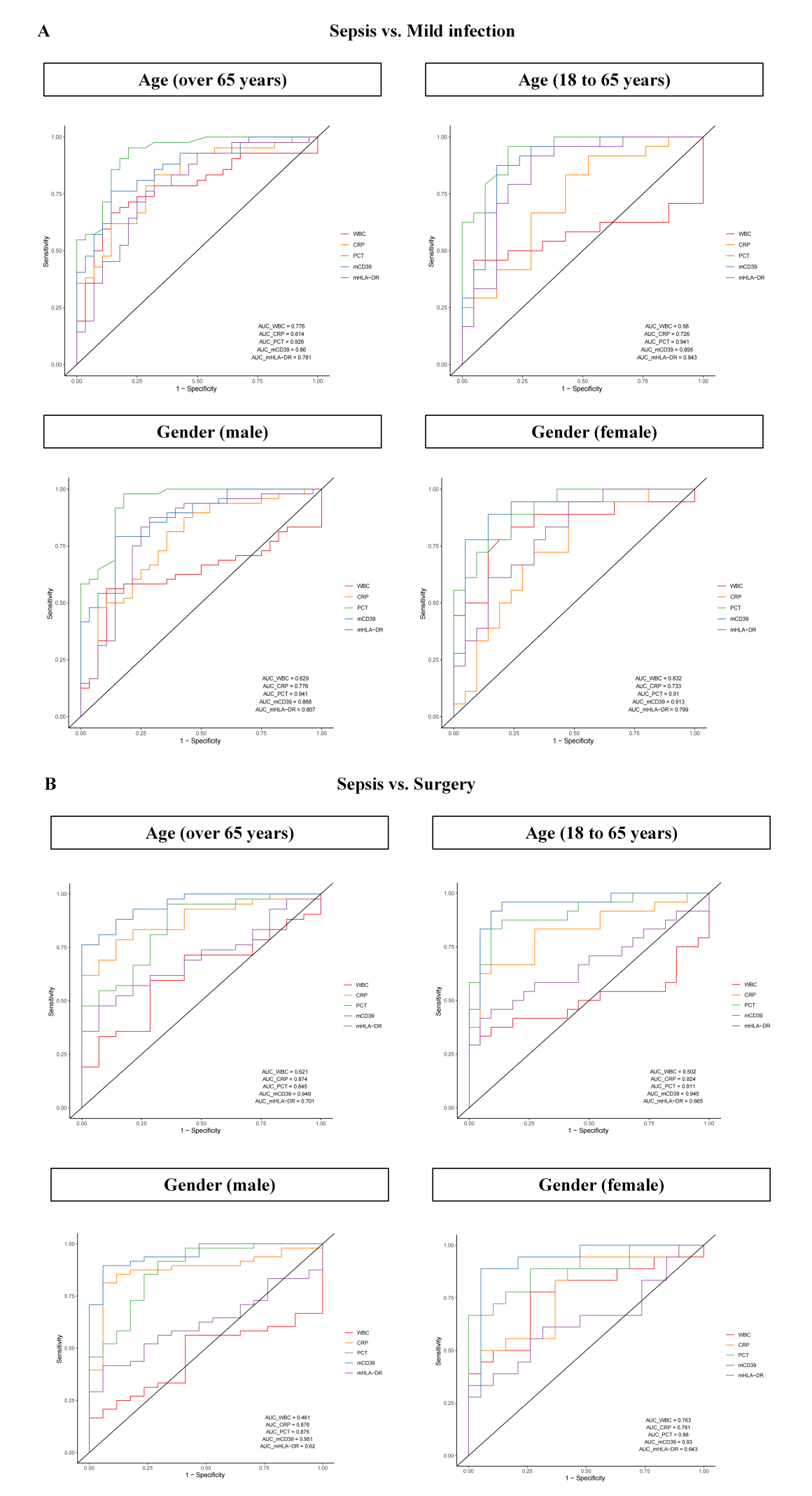
**

**Supplemental Fig. 3 The ROC curves displaying the capability of CD39 and commonly used biomarkers in diagnosis of sepsis patients stratified by age and gender.** After stratification by age and gender, ROC curves for WBC, CRP, PCT, mCD39, and mHLA-DR were utilized to examine their ability to distinguish patients between: **A** sepsis group and mild infection group; **B** sepsis group and surgery group.

ROC: receiver operating characteristic. WBC: white blood cell count. CRP: C-reactive protein. PCT: procalcitonin. mCD39: monocytic CD39. mHLA-DR: monocytic human leukocyte antigen-DR. AUC: area under the curve.

**
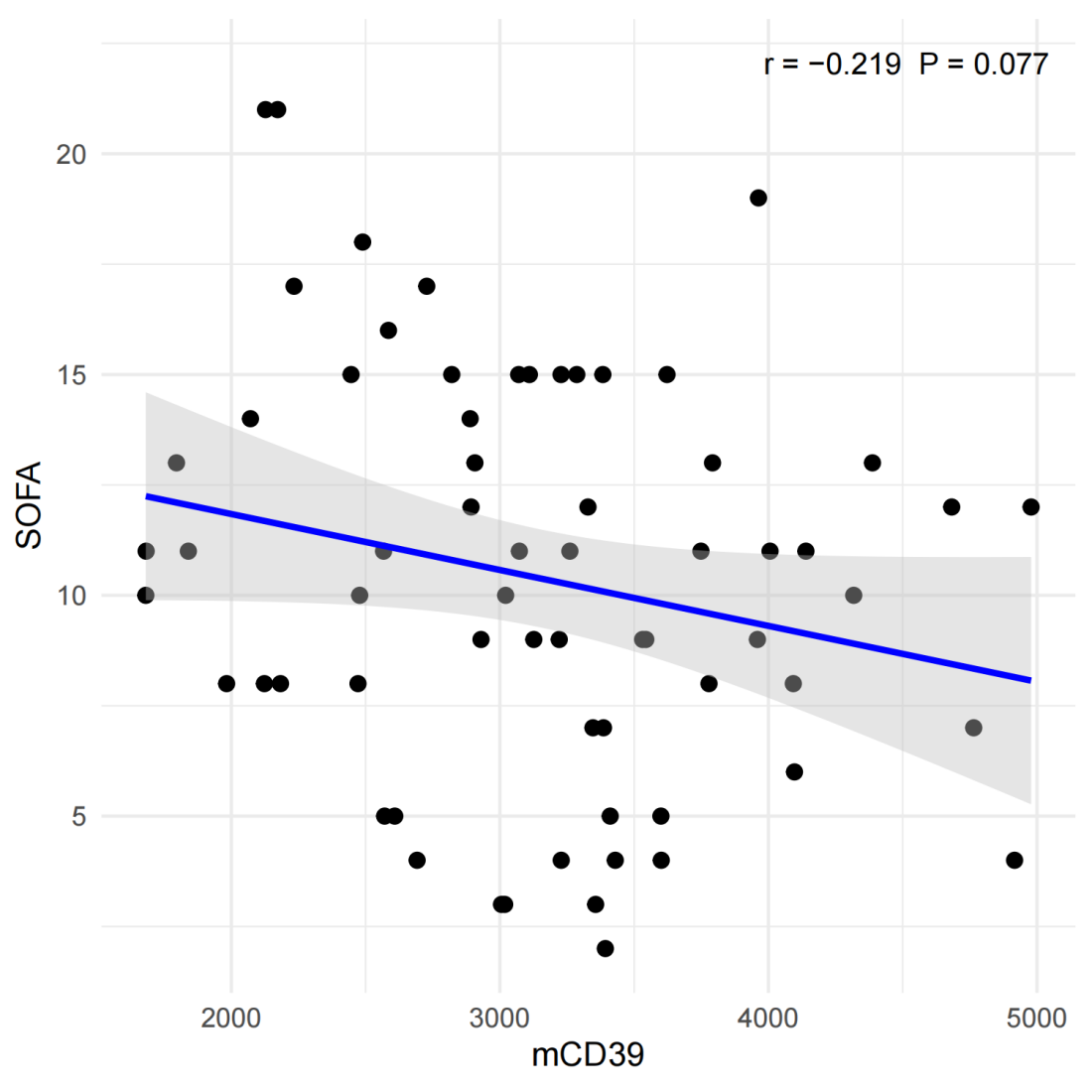
**

**Supplemental Fig. 4** Scatterplot of mCD39 vs. SOFA scores of sepsis group in the validation cohort.

mCD39: monocytic CD39. SOFA: Sequential Organ Failure Assessment.
